# Anaesthesia, not number of sessions, influences the magnitude and duration of an aHF-rTMS in dogs

**DOI:** 10.1371/journal.pone.0185362

**Published:** 2017-09-22

**Authors:** Robrecht Dockx, Kathelijne Peremans, Lise Vlerick, Nick Van Laeken, Jimmy H. Saunders, Ingeborgh Polis, Filip De Vos, Chris Baeken

**Affiliations:** 1 Ghent Experimental Psychiatry (GHEP) lab, Department of Psychiatry and Medical Psychology, Ghent University, Ghent, East Flanders, Belgium; 2 Department of Veterinary medical imaging and small animal orthopaedics, Faculty of Veterinary Medicine, Ghent University, Merelbeke, East Flanders, Belgium; 3 Laboratory of Radiopharmacy, Department of Pharmaceutical Analysis, Faculty of Pharmaceutical Sciences, Ghent University, Ghent, East Flanders, Begium; 4 Department of Small Animal, Faculty of Veterinary Medicine, Ghent University, Merelbeke, East Flanders, Belgium; Nanjing Normal University, CHINA

## Abstract

**Background:**

Currently, the rat has been a useful animal model in brain stimulation research. Nevertheless, extrapolating results from rodent repetitive Transcranial Magnetic Stimulation (rTMS) research to humans contains several hurdles. This suggests the desperate need for a large animal model in translational rTMS research. The dog would be a valid choice, not only due to the fact that humans and dogs share a neurophysiological background, but a similar neuropathological background as well.

**Hypothesis:**

In order to evaluate the feasibility of the canine rTMS animal model, this study aimed to evaluate the neurophysiological response in dogs on a, clinically used, accelerated high frequency (aHF) rTMS protocol. This aHF-rTMS (20 Hz) protocol was performed under anaesthesia or sedation and either 20 sessions or 5 sessions were given to each dog.

**Methods:**

21 healthy dogs were randomly subjected to one of the four aHF-rTMS protocols (1 sham and 3 active protocols). For each dog, the perfusion indices (PI), of a [^99m^Tc]HMPAO scan at 4 time points, for the left frontal cortex (stimulation target) were calculated for each protocol.

**Results:**

Concerning sham stimulation, the average PI remained at the baseline level. The main result was the presence of a direct transitory increase in rCBF at the stimulation site, both under anaesthesia and sedation. Nevertheless the measured increase in rCBF was higher but shorter duration under sedation. The magnitude of this increase was not influenced by number of sessions. No changes in rCBF were found in remote brain regions.

**Conclusion:**

This study shows that, despite the influence of anaesthesia and sedation, comparable and clinically relevant effects on the rCBF can be obtained in dogs. Since less methodological hurdles have to be overcome and comparable results can be obtained, it would be acceptable to put the dog forward as an alternative translational rTMS animal model.

## Introduction

Transcranial magnetic stimulation (TMS) is a non-invasive brain stimulation technique that relies on Faraday’s law of induction. An electric pulse is generated and is sent through a dense winding of conducting material, the stimulating coil, creating a magnetic field perpendicular to the pulse flow. When applied alternated over the head, electrical currents—with an opposite direction to the pulse flow—are induced in the superficial cortical areas [1, 2]. Repetitive TMS is used clinically, to treat major depression, anxiety disorders, addiction, Alzheimer disease, chronic pain, stroke, tinnitus, etc. Only a level A recommendation is given for the beneficial effects of High Frequency rTMS (HF-rTMS) in major depressive disorder (MDD) and neuropathic pain [2–5]. The stimulation in humans is applied while being conscious, this in contrast to (r)TMS in animals, where anaesthesia or sedation is required. The latter is applied for ethical and technical considerations (e.g. movement).

Despite the beneficial effects of HF-rTMS, there is still a desperate need for animal models in the field of rTMS. The current animal models are frequently used to (1) assess the basic mechanisms of rTMS, (2) evaluate the neurobiological changes induced by the magnetic field, (3) appraise its influence on neuropsychiatric disorders, and (4) examine the influence of the different stimulation parameters [1, 6]. Repetitive TMS research in rats has attributed a lot in unravelling its mechanism. This research has not only shown changes in monoamines [7–10], amino acids [11, 12] and Blood Derived Neurotrophic Factor (BDNF) [13, 14], but in behaviour and neuropsychiatric rat models as well [10, 15, 16]. Nonetheless, the rat model does not comply with human studies completely.

In order to mirror human TMS studies, accurate and focal stimulation in animals is essential. A human figure-of-eight coil can easily affect 100–200 mm^2^ of the underlying cortical area [17] and stimulate as focal as 0.5 cm^3^ [18] whereas the adult rat brain comprise on average only 1.5 cm^3^. [6]. This implies that while focal stimulation is achieved in humans, whole brain stimulations are executed in the rat [19]. A solution to achieve focal stimulation in rats is the use of smaller, more focal coils. Notwithstanding, a reduction in coil size involves limiting factors such as coil overheating and a drop in efficiency [17]. As important as focality is the accuracy of the coil placement. Localization of the target region in rats is frequently done by means of stereotactic frames, which supplies a larger level of accuracy than non-stereotactic frameless neuronavigation systems. Nonetheless, time, safety and cost can be reduced when choosing frameless over frame based systems [20]. It is clear that, when extrapolating rTMS research from rodents to humans, some hurdles must be overcome [6].

Although rTMS can be conducted in awake humans and animals, anaesthesia/sedation may be needed in animal models [19]. Stimulation under sedation and anaesthesia is preferred to stimulation while awake/mechanically restraint. While conscious, dogs might react on the acoustic and tactile stimuli provoked by the TMS, which could cause a loss of focality and efficacy. Despite the fact that anaesthesia (e.g. dexmedetomidine, isoflurane, midazolam, ketamine) depresses the neural activity [21–24], neural effects of rTMS have been shown in anesthetized rats. However, Gersner et al. (2011) found different effects on neuroplasticity markers (BDNF, GluR1) between anesthetized and awake rats after rTMS.

Recently cats, dogs and monkeys have been subjected to (r)TMS [25–32]. The use of these animals as model would allow accurate and focal stimulations in awake animals, mirroring human (r)TMS research. Although it has been possible to stimulate cats and monkeys awake [26, 28], anaesthesia or sedation may still be preferred [21]. Based on the phylogenetic closeness, the monkey would be the preferred animal model in preclinical rTMS research. Nevertheless, high costs and ethical considerations coincide with the use of monkeys, which limits the use of this species in preclinical research [6]. Besides monkeys, dogs have proven their ability to be a valid natural animal model for several psychiatric conditions [28, 31, 33–37]. While conscious, animals might react to the acoustic and tactile stimuli provoked by the TMS, which could cause a loss of focality and efficacy [6]. Therefore, this study aims to evaluate and compare the short and long-term effects, by means of changes in regional cerebral perfusion (Perfusion Index, PI), provoked by a navigated, accelerated, High Frequency (20 Hz) rTMS (aHF-rTMS) protocol over the left frontal cortex [38] in 21 healthy dogs. Two stimulation conditions are focussed on: the number of sessions (20 sessions vs 5 sessions) and the consciousness state (anaesthesia vs sedation). It was hypothesized that the PI, at the stimulation site would differ significantly (α = 0.05) for each stimulation condition. [^99m^Tc]HMPAO SPECT (Single Photon Emission Computed Tomography; d, 1, hexamethylpropylene amine oxime) scans were used to semi-quantify the perfusion indices (PI) of the cortical, cerebellar, subcortical areas and the olfactory bulb [39, 40].

## Materials and methods

### Animals

Twenty-one healthy neutered dogs (2 fox-hounds and 15 beagles; 13 males and 4 neutered females; aged between 3 and 8 years old) were incorporated in this study. For practical reasons, four out of 21 dogs were randomly selected for reuse. Only after a three-month washout period (equals 6 months after the last stimulation session) and a return to baseline perfusion index (measured by SPECT) the dogs were reused and considered as a new test subject. Hence, 21 (17 used and 4 reused) dogs entered the study. This study includes, mere for statistical comparison, own data extracted from Dockx et al (2015) [41, 42]. The guidelines for animal welfare, imposed by the ethical committee were respected. This study (EC 2015_38) was approved by The Ghent University Ethical Committee. The dogs were provided by the department of veterinary medical imaging and small animal orthopedics and the department small animals of the faculty of veterinary medicine. The dogs were permanently housed in groups (newly built housing since this year in the new farm of small pets) of 8 on an internal surface of 15 m^2^ with permanent access to an outside area of 15 m^2^. The floor coverings in the inner part consisted of wood shavings. Frequently, toys such as Kongs® were given to the animals and were twice a day released onto an enclosed play area. In addition, the dogs were regularly walked by students of the faculty of veterinary medicine. After the HMPAO scans, the dogs stayed 1 night at the Nuclear Veterinary Department, where they were accommodated in a separate 3.5 x 3.7 meter cage. No animals were sacrifised at the end of this study. A sample size calculation was performed based on a prediction linear mixed model with a delta (predicated difference) equal to 0.05 and a power of 0.80. This provided a sample size of 7.48 animals per group. Therefore, a sample size of 8 animals per group was chosen.

### Neuronavigation protocol

In order to perform the neuronavigation, a tomographical dataset was required. Each dog underwent a magnetic resonance imaging (MRI) scan. This acquisition was performed by a Siemens 3T Magnetom Trio Tim system (Siemens Medical Systems, Erlangen, Germany). A phased-array spine coil and a phased-array body matrix coil were used to obtain the data set. A T1-weighted 3D MPRAGE sequence with 176 sagittal slices was acquired. The following sequence parameters were used: TR = 2250 ms, TE = 4,18 ms, TI = 900 ms, parallel acquisition method = GRAPPA with acceleration factor = 2, matrix size = 256 ~ 256, sagittal, FOV = 220 mm, flip angle = 9°, voxel size = 0.9 ~ 0.86 ~ 0.86 mm3.

After the placement of an intravenous cephalic catheter, the dogs were intramuscularly (IM) premedicated with dexmedetomidine 375 μg/m^2^ body surface, Dexdomitor®, Orion Corporation, Espoo, Finland). General anaesthesia was achieved by an intravenous (IV) propofol injection (Propovet Multidose®, Abbott Laboratories, Berkshire, UK, 1–2 mg/kg given to effect) and maintained with isoflurane (Isoflo®, Abbott Laboratories, Berkshire, UK) in oxygen through a rebreathing system.

During the MRI acquisition the dogs were sternally positioned, head first in the scanner bore. The neuronavigation was performed while the dogs were recovering from general anaesthesia. When needed, the dogs were given dexmedetomidine (0,5–1 μg/kg to effect; IV) to finish the neuronavigation. The dogs’ heads were fixated in a self-made mould and the subject tracker was attached to the neck region.

After the data were loaded into the software (Brainsight, Rogue-resolutions Ltd, Cardiff, UK) a skin reconstruction was made, on which three to four fiducial markers (landmarks) were set and identified. The left frontal cortex was targeted by manually identifying its centre on the MRI data set. The target’s external position was located by holding a pointer–connected to three reflecting balls—perpendicular over the target region as indicated by the neuronavigation software (for a full description we refer to Dockx et al. (2017, accepted for publication in PeerJ) [43].

### The stimulation protocol

The 21 dogs were randomly divided into 3 unequal groups: group 1 (n = 5), group 2 (n = 8) and group 3 (n = 8). Five neutered male beagle of 6.6 years (sd = 2.01) and 8 neutered beagles (2 females, 6 males) of 5.75 years old (sd = 2.14) were included in group 1 respectively group 2. Group 3 comprised 2 male neutered foxhounds and 6 (3 females, 3 males) beagle dogs (all neutered) with an average age of 6.25 years (sd = 0.98).

The data extracted from Dockx et al. (2015) was derived from 8 neutered dogs, group 4. This group consisted of 6 beagles and 2 mix breed foxhounds (4 males, 4 females; aged between 4 and 8 years).

By using positive reinforcement techniques, all dogs were accustomed to the researchers, the stimulating room and the sound and placing of a sham coil. This was done several months before the start of the stimulation experiment. For cardiovascular reasons, it was chosen to perform all stimulation protocols in groups 1, 2 and 3 under general anaesthesia. Premedication consisted of butorphanol IV (0.2 mg/kg; Dolorex®; Intervet Belgium NV). After onset of sedation anaesthesia was induced intravenously by administering midazolam (0.2 mg/kg; Dormicum®; Roche Nederland B.V.) immediately followed by propofol (Propovet Multidose®, Abbott Laboratories, Berkshire, UK, 1–2 mg/kg given to effect). General anaesthesia was maintained with isoflurane (Isoflo®, Abbott Laboratories, Berkshire, UK) in oxygen using a rebreathing system. Group 4 underwent the stimulation protocol under IM sedation with dexmedetomidine at 375 μg/m^2^ body surface. When deemed necessary an additional dose of 183 μg/m^2^ was injected intramuscularly.

Immediately following the induction of anaesthesia/sedation, the motor threshold of the left motor cortex was determined. A motor threshold (MT) of 100% was defined as the set machine output (Magstim Company Limited, Wales, UK) that could provoke 5 out of 10 visible muscle contractions in the right upper front limb. After the assessment of the MT, the external localisation of the centre of the left frontal cortex was located based on the previously measured X,Y positions and marked with a permanent marker on the fur. The centre of a standard figure-of-eight coil was placed perpendicular over the mark with the handle pointing abaxial. For the sham group, the coil was placed in a 90-degree angle with one wing making contact with the skull. HF-rTMS protocol (20Hz, 110% MT) was applied to the left frontal cortex. The animals received, based on the group they were divided into, 20 sham sessions under general anaesthesia (group 1; 5 daily sessions during 4 days), 5 active sessions under anaesthesia or sedation on 1 day (group 2 and group 4 respectively) or 20 active sessions under general anaesthesia (group 3; 5 daily sessions during 4 days). Each session contained 40 trains of 1.9 seconds each. The trains were separated by a 12 second intertrain interval (in total 1560 pulses were given per session). The time interval between sessions was 10 to 15 minutes. This protocol was an exact copy of an accelerated HF-rTMS treatment protocol performed in MDD patients at our medial university hospital [44, 45]. The depth of the anaesthesia during each stage of the study was monitored by an anaesthesiologist. In order to maintain a constant anaesthesia depth, clinical parameters (such as ventral position of the eye and absence of the eyelid-reflex) were used in combination with a constant end-tidal isoflurane concentration. When deemed necessary, the isoflurane dose was adjusted to maintain the same depth. Under sedation, each rTMS session was preceded and followed by checking the sedation depth. Only when the dogs were not responsive to external stimuli, the next session could be applied.

### Tracer

Less than 24 hours prior to each SPECT scan a ^99^Mo generator was eluted. Approximately 1,85 GBq ^99m^TcO_4_ was added to the exametazime (d,1 hexamethylpropylene amine oxime (HMPAO); Ceretec®, GE Healthcare LTD, UK).

### SPECT scanning procedure

Prior to the stimulation sessions, each dog received a baseline [^99m^Tc]HMPAO-SPECT scan. After the last stimulation session, the dogs received 3 additional HMPAO-SPECT scans: 24 hours, 1 month and 3 months post stimulation. In order to perform these scans, an intravenous cephalic catheter was placed and the dogs were IM pre-medicated with dexmedetomidine (375μg/m2 body surface). When sedated, the dogs were given 348,54 MBq (sd 26,64 MBq)99mTc-HMPAO IV. Induction of anaesthesia was achieved 15–20 minutes after the tracer injection by administering propofol IV (1–2 mg/kg body weight to effect). Again, general anesthesia was maintained with isoflurane in oxygen through a rebreathing system. Respiratory and electrocardiographic monitoring was used during the entire duration of each scan. Equipped with low energy ultrahigh-resolution parallel hole collimators (tomographic resolution FWHM = 9 mm), a triple head gamma camera (Triad, Trionix, Twinsburg, OH, USA) was used 30–35 minutes after the tracer injection to acquire the data. The camera collected data over a circular 360° rotation in a step-and-shoot mode during 20 minutes (120 steps, 10 sec per step, 3° steps) on a 128~128 matrix. Afterwards, the data were iteratively reconstructed and a Butterworth filter (cut-off 1.4 cycli/cm, order 5) was added.

### Image analysis

A template containing 11 fixed, different brain regions (both frontal, temporal, parietal and occipital lobes, the cerebellum, olfactory bulb and the subcortical area) was, using BRASS software (Brain Registration and Automated SPECT Semiquantification, Nuclear diagnostics, Sweden), fitted onto each SPECT scan. This template, composed from 14 dogs (9 male, 5 female, mean age 50 months ± 20), eliminates the operator dependent demarcation of the volumes of interest (VOI). Hereby facilitating the fitting procedure that is necessary to compensate for inter-individual differences in brain size and shape. The regional Cerebral Blood Flow (rCBF; perfusion index (PI)) was automatically calculated for each individual dog by normalizing the regional radioactivity to the radioactivity of the entire brain. The left frontal cortex was of major interest but since the PIs of the other 10 regions were automatically calculated, these data were also included in the analysis.

### Statistical analysis

Rstudio 1.0.136 (R: A Language and Environment for Statistical Computing; R Core Team; R Foundation for Statistical Computing, Vienna, Austria, 2016, https://www.R-project.org/) with package nlme version 3.1–131 was used to compute all analyses.

At first, a simple main effects model was fitted with PI as outcome variable and time point and treatment group set as fixed effects. Forward stepwise regressions model building was used (α_in_ = 0.1, α_out_ = 0.15). During the model building process, multicollinearity was taken into account. After identifying the main effects, the interactions between the different variables were assessed (α_in_ = α_out_ = 0.05).

For the first dataset, PI of the left frontal cortex was determined at 4 time points (baseline, 24 hours post, 1 month and 3 months) after stimulation under general anaesthesia. The primary objective was to compare PI at each time point between and within the treatment groups: 20 sessions sham stimulation as reference, and 5 vs. 20 sessions of active stimulation. A linear mixed model was fitted on this response variable with treatment group and time points as fixed-effect factors and subjects as random effect to account for correlations between repeated measurements. A random slope was included in the model to account for a change in variance over time. The presence of a time point by treatment interaction was also considered in the model. Gender and age were considered as fixed effect. Post hoc, this model was fitted onto the PI of the remaining 10 VOI in order to detect any distant effects of the stimulation protocol.

For comparing aHF-rTMS stimulation under general anaesthesia and sedation, a previous dataset (Dockx et al., 2015) was used, where PI of the left frontal cortex was determined by SPECT scan at the same 4 time points after 5 active sessions under sedation (group 4). The model is the same as the previous one, except that the treatment groups are now stimulation under sedation and anaesthesia (always 5 active sessions).

The significance level of was set at 0.05, two-tailed. The assumptions of normality of the error terms, linearity of the regression function, homoscedasticity and independence of the error term were checked based on diagnostics plots combined with statistical tests (Bartlett test of homogeneity of variances and the Shapiro-Wilk normality test).

## Results

The set model assumptions, with the exception of independence due to the repeated measures, were met based on the diagnostics plots. The Bartlett test of homogeneity and the Shapiro-Wilk normality test revealed a p-value larger than the pre-set significance level of 0.05, two-tailed. No outliers were detected. The variables “gender” and “age” did not have a significant influence on the outcome variable and were thus removed from the model.

### The influence of the number of sessions on the short and long term effects of an aHF-rTMS protocol under anaesthesia

The fitted model was written as E(Yt|T_1_,T_2_) = β_0_ + β_1_t_1_ + β_2_t_2_ + β_3_t_3_ + β_4_T_1_ + β_5_T_2_ + β_6_t_1_T_1_ + β_7_t_2_T_1_+ β_8_t_3_T_1_+ β_9_t_1_T_2_+ β_10_t_2_T_2_+ β_11_t_3_T_2_ with Yt (PI left frontal cortex) as response variable. The first predictor value t denoted the different SPECT scan time points, t1 the first of three (k-1 = 4–1 = 3) dummies (= 1 if time point = “24 hours post” or 0 otherwise), t2 the second dummy (= 1 if time point = “1 month post” or 0 otherwise) and t3 (= 1 if time point = “3 months post” or 0 otherwise). With T denoting the different stimulation conditions (treatment variable, categorical), T1 the first of 2 dummies (= 1 if the stimulation protocol = “5 active sessions under anaesthesia” or 0 otherwise) and T2 (= 1 if the stimulation protocol = “20 active sessions under anaesthesia” or 0 otherwise).

Based on Table 1 there is a significant influence (*p* < 0.05) of the treatment group on the average PI of the left frontal cortex when compared to the baseline PI of the first group (20 sham sessions under anaesthesia), whereas time points showed no main effect. Based on the set model, contrasts were created within each treatment group and time point.

**Table 1:** Output type III main effect test for the stimulation protocols under anaesthesia.

	Model 1 output		
	*numDF*	*F-value*	*p-value*
**(Intercept)**	1	7130.60	<0.001**
**Time point**	3	0.11	0.95
**Treatment group**	2	3.58	0.03*
**Interaction**	6	1.34	0.25

* < 0.05

**<0.001

**Fig 1:**
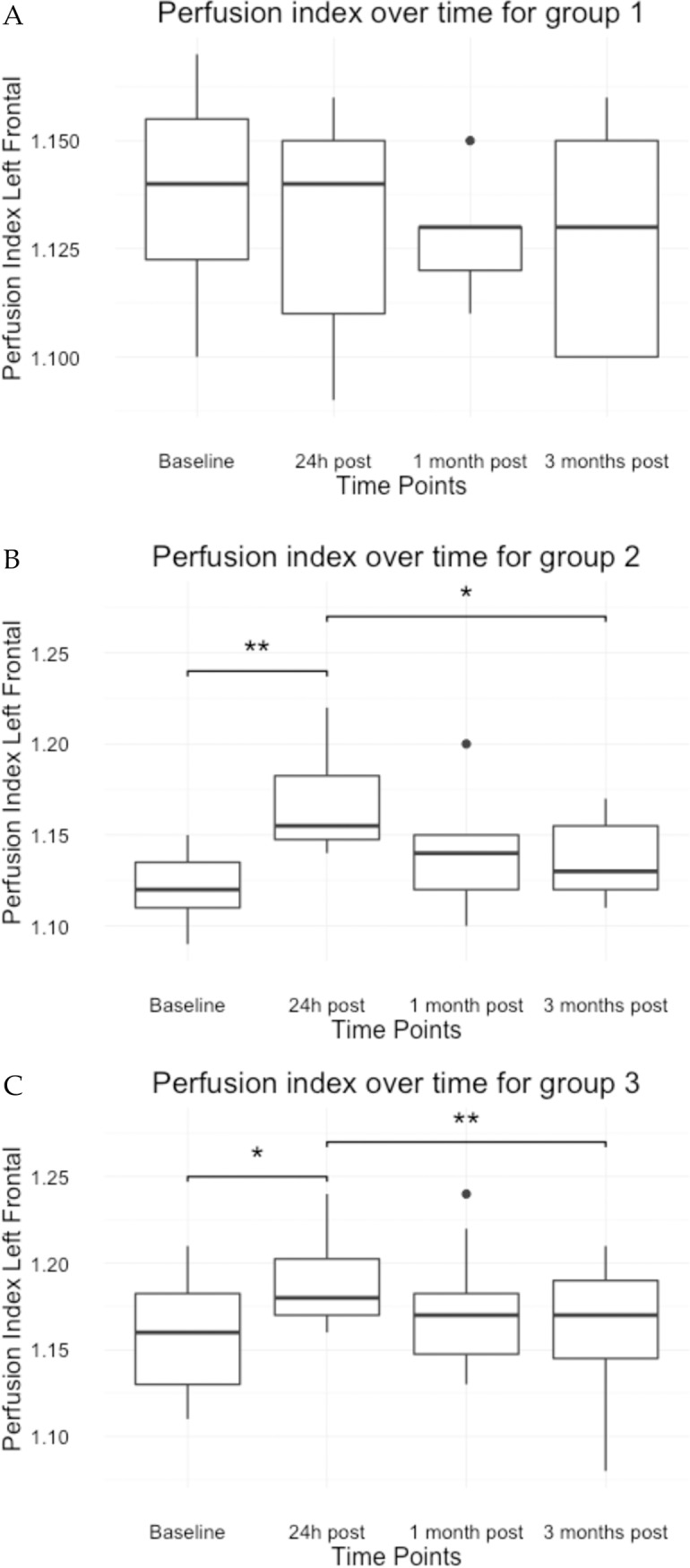
Boxplots of the left frontal cortex perfusion index for each treatment group based on the first model. (A = group 1; B = group 2; C = group 3; * < 0.01; **<0.001).

Within group 1 (20 sham sessions under anaesthesia) no significant differences in average PI were found between the different time points (Fig 1). Group 2 and 3 had an average PI at 24 hours post stimulation that differed significantly from the baseline PI as well as from the PI at 3 months after the last stimulation session (Table 2, Fig 1).

**Table 2:** Multiple comparison for each time point within the active stimulation protocols.

	5 active sessions	20 active sessions
	*p-value*	*p-value*
**Baseline—24h post**	<0.001**	0.009*
**Baseline—1 month post**	0.267	0.320
**Baseline—3 months post**	0.129	0.978
**24h post—1 month post**	0.074	0.247
**24h post—3 months post**	0.004*	0.001**
**1 month post—3 months post**	0.913	0.288

* < 0.01

**<0.001

**Fig 2:**
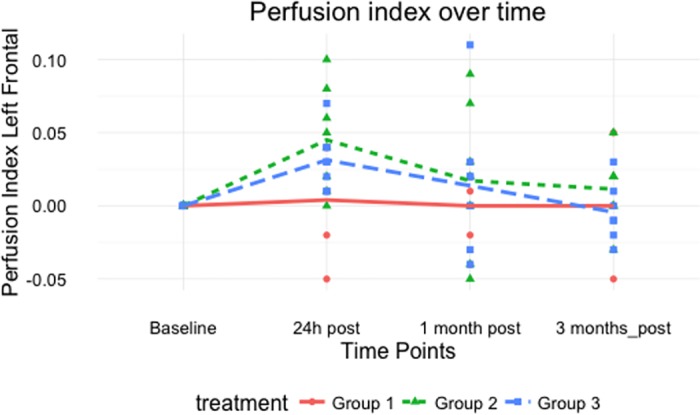
Difference in perfusion index of the left frontal cortex for the three treatment groups under anaesthesia.

Between the treatment groups, differences in PI were found 24h post and 1 month after the last stimulation session. At baseline no differences in average PI were found between the active stimulation protocols and the sham stimulation, whereas both active stimulation protocols differed significantly with the sham protocol at 24 hours post stimulation. At the given time point the average PI increased, compared to the reference level, 0.050 (95% CI -0.003; 0.102) and 0.039 (95% CI -0.016; 0.090) in group 2 respectively group 3. Between the two active stimulation protocols (1 day versus 5 days under anaesthesia), there was no significant difference between the average PI at the 24 hours’ time. One month post stimulation there was no difference in average PI between group 1 and 2. Nonetheless a significant difference was found between group 1 and group 3 (p = 0.014) and between group 2 and group 3 (p = 0.042). Three months after the last stimulation session, no significant differences were found over all stimulation groups. As seen in S1 Fig, each dog can react differently on the stimulation. Nonetheless, an average transient increase for each active protocol is present (Fig 2).

Post hoc the statistical model showed no changes in average PI for the brain regions other than the left frontal cortex, compared to the reference level (S3 Fig).

### The influence of depth of anaesthesia on the [46]short and long term effects of a single day aHF-rTMS protocol

Similar to the previous model, the results indicate differences in PI between and within each treatment group. The intercept was set at the baseline time point of the 5 active sessions stimulation protocol under anaesthesia. The fitted model was written as E(Yt|T1) = β_0_ + β_1_t_1_ + β_2_t_2_ + β_3_T_1_ + β_4_t_1_T_1_ + β_5_t_2_T_1_ with Yt (PI left frontal cortex) as response variable. The first predictor value t denoted the different SPECT scan time points, t_1_ the first of three (k-1 = 3–1 = 2) dummies (= 1 if time point = “24 hours post” or 0 otherwise) and t_2_ the second dummy (= 1 if time point = “3 month post” or 0 otherwise. With T denoting the stimulation protocol (treatment variable, categorical), T_1_ the only dummy (= 1 if the stimulation protocol = “5 active sessions under sedation” or 0 otherwise). The presence of a time point by stimulation protocol interaction was considered in the model. By default, the first level of the time point and stimulation protocol group was coded as the reference level (baseline and 5 active stimulation sessions under anaesthesia).

**Fig 3:**
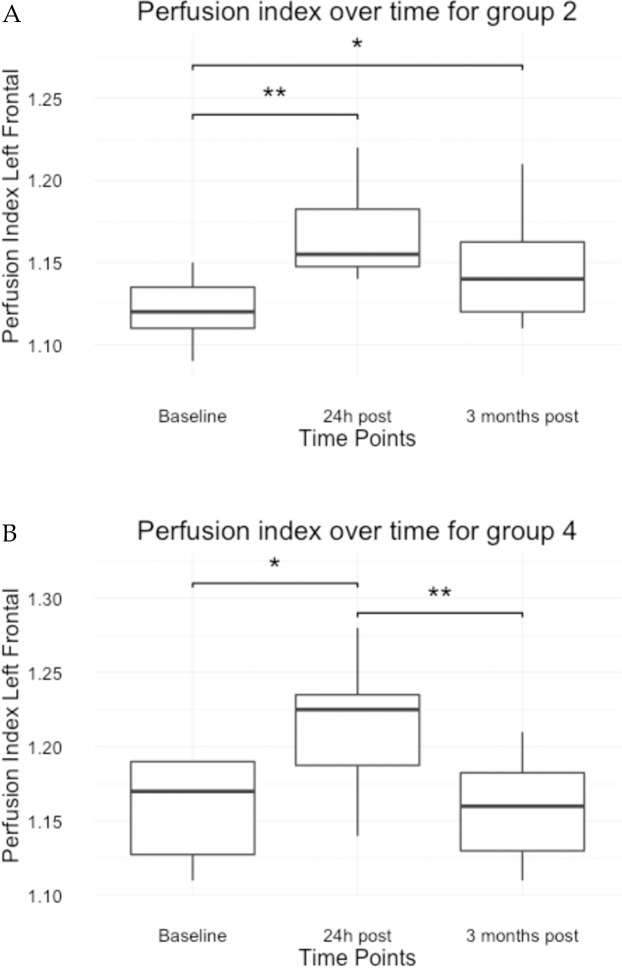
Boxplots of the left frontal cortex perfusion index for each treatment group based on the second model. **(**A = group 2; B = group 4; * < 0.05; **<0.01;).

**Fig 4:**
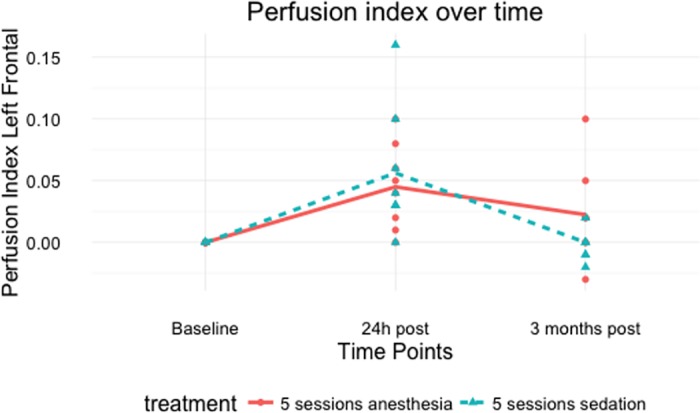
Difference in perfusion index of the left frontal cortex for the two treatment groups that underwent 5 aHF-rTMS sessions.

The two stimulation protocols differed from each other (*p*-value = 0.009). Table 3 indicates that the effect of the stimulation is again present at the stimulation site for both protocols but that the effect last longer under anaesthesia. Even more, a significant difference of 0.036 (95%CI 0.009; 0.064) was found between the average PI at time point 24h post whereas this difference was not present at time point 3 months post stimulation under sedation. The PI returned to baseline after 3 months under sedation whereas it did not under anaesthesia (Fig 3, Table 4). As seen in S2 Fig each dog can react differently on the stimulation. Nonetheless, an average transient increase for each active protocol is present (Fig 4).

**Table 3:** Output type III main effect test for the 5 session stimulation protocols.

	Model 2 output		
	*numDF*	*F-value*	*p-value*
**(Intercept)**	1	12785.57	<0.001**
**Time point**	2	5.19	0.010*
**Treatment group**	1	6.67	0.013*
**Interaction**	2	1.82	0.175

* < 0.05

**<0.001

**Table 4:** Multiple comparison for each time point within the 5 active sessions stimulation protocols.

	5 active session anaesthesia	5 active sessions sedation
	*p-value*	*p-value*
**Baseline—24h post**	0.007**	0.001**
**Baseline—3 months post**	0.030*	1.000
**24h post—3 months post**	0.649	0.001**

* < 0.05

**<0.01

Post hoc the statistical model showed no changes in average PI for the brain regions other than the left frontal cortex, compared to the reference level (S4 Fig).

## Discussion

Accelerated HF-rTMS—delivered over the left frontal cortex—was applied one or four consecutive days in sedated or anesthetized dogs. In line with comparable research in depressed patients, sham stimulation did not show short or long term effects on cerebral perfusion [46–48]. Active aHF-rTMS resulted in a transitory increase in rCBF at the stimulation site, under anaesthesia as well as under sedation [49]. Although the magnitude of this increase was not influenced by the number of sessions, the increase in rCBF was higher but more short-lived under sedation. Under anaesthesia, both active protocols (5 and 20 sessions aHF-rTMS) provoked increased neuronal activity in the left frontal cortex, which lasted until 1 month after stimulation. This is comparable with the work executed in human subjects, undergoing 10 consecutive daily HF-rTMS sessions (one session/day), that observed an increased neuronal activity lasting up to 2 weeks post stimulation [46, 47, 50]. Moreover, this study obtained a comparable increase in rCBF of the left frontal cortex (2–3%) as Catafau et al (2001) reported in 7 depressed patients (medication-resistant). Although we did not assess behavioural measurements, this observed perfusion increase might initiate speculation on the potential role of rTMS in behaviour-disordered dogs, especially in anxiety disordered animals that have been reported to suffer from hypoperfusion in the left frontal cortex [35]. Similarly, increased rCBF in the left frontal region after HF-rTMS has been associated with clinical improvement in MDD patients [51].

In this study, we only detected increased rCBF after active aHF-rTMS limited to the stimulated area, and not more widespread in the structurally and functionally connected areas. A reason for the absence of remote effects could be the state in which the animals were stimulated. A study on human subject by Massimini et al. (2005) found that during non-rapid eye movement sleep (NREM) the initial response at stimulation site did not propagate to distant regions. They explained this by a loss of cortical integration during NREM sleep, which also occurs during midazolam-induced loss of consciousness [52, 53]. Ferrarelli et al. (2010) found an hd-EEG response with a short lasting high positive-negative wave under midazolam anaesthesia indicating a local and shorter TMS activity in contrast to the effect registered during wakefulness. Aside from the effects of midazolam, all volatile anaesthetics can affect neuroplasticity, reduce excitatory and augment inhibitory neuronal transmission. In conclusion, it is therefore possible that the absence of remote activation is confounded by the combined use of midazolam and isoflurane.

Despite the presence of a (linear) dose-response relationship [54–56], the measured significant increase in rCBF after stimulation was identical for the 2 active protocols (5 session or 20 sessions) under anaesthesia. This implies that an increase in number of sessions does not seem to influence the magnitude of the rCBF increase. Nonetheless, this does not rule out the presence of a (linear) dose-response relationship. In this study, it was not possible to determine a response rate, which excludes the possibility to examine the presence of a dose-response relationship.

Although the descriptive analysis of this study revealed an individual response to the aHF-rTMS protocol (Figs 3 and 4), no main effect was found for the fixed variables age and gender. This is in line with studies performed in human subjects that found that gender and age were no significant predictor variables for the outcome of an rTMS treatment [51, 57–60]. Nonetheless, a better rTMS outcome has been found in younger people [61] and rats [62]. It is hypothesized that in older individuals the distance between the frontal cortex and the skull increases increase, limiting the rTMS response. Since aging also causes atrophy of the canine brain [63], it is plausible that the canine cortex-scalp distance increases as well, thus provoking an age related response. In the current study, no age main effect was found, due to the selection of an age homogeneous population. Although a gender dependent rTMS response is not present, it appears that a high oestradiol to progesterone ratio may positively influence the outcome of SSRI’s and rTMS treatment. [64]. This study used neutered female dogs, suggesting a lower oestradiol to progesterone ratio and therefor a lower rTMS response compared to intact female dogs.

When using a coil that provides a larger coil to brain size, the focality and efficacy might plummet [19]. Nonetheless, focality can be assumed in this study since only an increase in rCBF was found for the stimulation target and not in surrounding cortical regions. A focal increase in rCBF, might indicate that no whole brain stimulation took place notwithstanding the presence of a larger coil to brain size ration. The, in comparison, smaller canine brain could not have been able to capture the total flux generated by the coil, which might help to explain the absence of remote effects in this study.

Although this study has some major advantages such as including individual neuronavigation, some limitations must be kept in mind. An active coil, tilted 90 degrees was used as the sham condition. Despite the fact that an active coil, held this way, can provoke minor voltages in the underlying cortical tissue [65]. However, no changes in the rCBF were noticed in the control group. Only the one-day protocol under sedation was explored, leaving the question whether accelerated HF-rTMS under sedation allows propagation of the initial response at the stimulation site, unanswered. In the current study, 21 neutered healthy dogs were included. More information is needed regarding the effects of the gonadal status, age and natural brain disorders on the neuromodulation of the accelerated HF-rTMS protocols. Hereby, clearer insights into the canine rTMS model can be obtained and its use as a valid translational model for rTMS research. Nonetheless, it must be emphasised that the obtained results should be interpreted with caution. In order to exclude the effects of anaesthesia in the canine rTMS model; different anaesthetic/sedative protocols should be compared to conscious dogs while rTMS is applied. The final limitation is that SPECT has in comparison to PET a lower sensitivity.

To conclude, the results in this study suggest that accelerated HF-rTMS can provoke the neuronal activation in the stimulated cortical region in healthy anesthetized dogs. Because these findings—acquired with human rTMS apparatus—in healthy dogs strongly resemble SPECT findings in humans, is it reasonable to reserve a role for the dog as an alternative animal model for rTMS research in humans.

## Supporting information

S1 Fig Line plot for each individual dog in each treatment group. (TIF)

S2 Fig Line plot for each individual dog in each treatment group. (TIF)

S3 Fig Average perfusion indices for 10 brain regions for each time point for each treatment group under anaesthesia. (TIF)

S4 Fig Average perfusion indices for 10 brain regions for each time point for each treatment group that received 5 aHF-rTMS sessions. (TIF)

S5 Fig Average perfusion index of the left frontal cortex for the three treatment groups under anaesthesia. (TIF)

S6 Fig Average perfusion index of the left frontal cortex for the two treatment groups that underwent 5 aHF-rTMS sessions. (TIF)

S1 Table Descriptive statistics for the left frontal cortex perfusion index for each treatment group under anaesthesia (* only male beagle dogs were included assigned to this group). (DOCX)

S2 Table Descriptive statistics for the left frontal cortex perfusion index for each treatment group that underwent 5 aHF-rTMS sessions. (DOCX)

## References

[1] George MS, Lisanby SH, Sackeim HA. Transcranial magnetic stimulation—Applications in neuropsychiatry. Arch Gen Psychiat. 1999;56(4):300–11. doi: 10.1001/archpsyc.56.4.300 10197824 10.1001/archpsyc.56.4.300

[2] Lefaucheur J-P, Andre-Obadia N, Antal A, Ayache SS, Baeken C, Benninger DH, et al Evidence-based guidelines on the therapeutic use of repetitive transcranial magnetic stimulation (rTMS). Clinical Neurophysiology. 2014;125(11):2150–206. doi: 10.1016/j.clinph.2014.05.021 25034472 10.1016/j.clinph.2014.05.021

[3] Diefenbach GJ, Bragdon L, Goethe JW. Treating anxious depression using repetitive transcranial magnetic stimulation. Journal of affective disorders. 2013;151(1):365–8. doi: 10.1016/j.jad.2013.05.094 . 23810361 10.1016/j.jad.2013.05.094

[4] Shen Y, Cao XY, Tan T, Shan CL, Wang YJ, Pan JB, et al 10-Hz Repetitive Transcranial Magnetic Stimulation of the Left Dorsolateral Prefrontal Cortex Reduces Heroin Cue Craving in Long-Term Addicts. Biological psychiatry. 2016;80(3):E13–E4. doi: 10.1016/j.biopsych.2016.02.006 26995024 10.1016/j.biopsych.2016.02.006

[5] Terraneo A, Leggio L, Saladini M, Ermani M, Bonci A, Gallimberti L. Transcranial magnetic stimulation of dorsolateral prefrontal cortex reduces cocaine use: A pilot study. Eur Neuropsychopharm. 2016;26(1):37–44. doi: 10.1016/j.euroneuro.2015.11.011 26655188 10.1016/j.euroneuro.2015.11.011 PMC9379076

[6] Vahabzadeh-Hagh AM, Muller PA, Gersner R, Zangen A, Rotenberg A. Translational Neuromodulation: Approximating Human Transcranial Magnetic Stimulation Protocols in Rats. Neuromodulation. 2012;15(4):296–305. doi: 10.1111/j.1525-1403.2012.00482.x 22780329 10.1111/j.1525-1403.2012.00482.x PMC5764706

[7] Ben-Shachar D, Belmaker RH, Grisaru N, Klein E. Transcranial magnetic stimulation induces alterations in brain monoamines. J Neural Transm. 1997;104(2–3):191–7. doi: 10.1007/BF01273180 . 9203081 10.1007/BF01273180

[8] Ben-Shachar D, Gazawi H, Riboyad-Levin J, Klein E. Chronic repetitive transcranial magnetic stimulation alters beta-adrenergic and 5-HT2 receptor characteristics in rat brain. Brain research. 1999;816(1):78–83. doi: 10.1016/S0006-8993(98)01119-6 9878693 10.1016/s0006-8993(98)01119-6

[9] Keck ME, Engelmann M, Muller MB, Henniger MS, Hermann B, Rupprecht R, et al Repetitive transcranial magnetic stimulation induces active coping strategies and attenuates the neuroendocrine stress response in rats. J Psychiatr Res. 2000;34(4–5):265–76. . 11104838 10.1016/s0022-3956(00)00028-5

[10] Kanno M, Matsumoto M, Togashi H, Yoshioka M, Mano Y. Effects of acute repetitive transcranial magnetic stimulation on dopamine release in the rat dorsolateral striatum. J Neurol Sci. 2004;217(1):73–81. doi: 10.1016/j.jns.2003.08.013 14675613 10.1016/j.jns.2003.08.013

[11] Keck ME, Sillaber I, Ebner K, Welt T, Toschi N, Kaehler ST, et al Acute transcranial magnetic stimulation of frontal brain regions selectively modulates the release of vasopressin, biogenic amines and amino acids in the rat brain. The European journal of neuroscience. 2000;12(10):3713–20. . 11029641 10.1046/j.1460-9568.2000.00243.x

[12] Yue L, Xiao-Lin H, Tao S. The effects of chronic repetitive transcranial magnetic stimulation on glutamate and gamma-aminobutyric acid in rat brain. Brain research. 2009;1260:94–9. doi: 10.1016/j.brainres.2009.01.009 19401169 10.1016/j.brainres.2009.01.009

[13] Wang HY, Crupi D, Liu J, Stucky A, Cruciata G, Di Rocco A, et al Repetitive transcranial magnetic stimulation enhances BDNF-TrkB signaling in both brain and lymphocyte. The Journal of neuroscience: the official journal of the Society for Neuroscience. 2011;31(30):11044–54. doi: 10.1523/JNEUROSCI.2125-11.2011 ; PubMed Central PMCID: PMCPMC3161730. 21795553 10.1523/JNEUROSCI.2125-11.2011 PMC3161730

[14] Muller MB, Toschi N, Kresse AE, Post A, Keck ME. Long-term repetitive transcranial magnetic stimulation increases the expression of brain-derived neurotrophic factor and cholecystokinin mRNA, but not neuropeptide tyrosine mRNA in specific areas of rat brain. Neuropsychopharmacology: official publication of the American College of Neuropsychopharmacology. 2000;23(2):205–15. doi: 10.1016/S0893-133x(00)00099-3 10882847 10.1016/S0893-133X(00)00099-3

[15] Sachdev PS, McBride R, Loo C, Mitchell PM, Malhi GS, Croker V. Effects of different frequencies of transcranial magnetic stimulation (TMS) on the forced swim test model of depression in rats. Biological psychiatry. 2002;51(6):474–9. . 11922882 10.1016/s0006-3223(01)01298-7

[16] Fleischmann A, Prolov K, Abarbanel J, Belmaker RH. The Effect of Transcranial Magnetic Stimulation of Rat-Brain on Behavioral-Models of Depression. Brain research. 1995;699(1):130–2. doi: 10.1016/0006-8993(95)01018-Q 8616602 10.1016/0006-8993(95)01018-q

[17] Wagner T, Rushmore J, Eden U, Valero-Cabre A. Biophysical foundations underlying TMS: Setting the stage for an effective use of neurostimulation in the cognitive neurosciences. Cortex. 2009;45(9):1025–34. doi: 10.1016/j.cortex.2008.10.002 19027896 10.1016/j.cortex.2008.10.002 PMC3417820

[18] Roth Y, Amir A, Levkovitz Y, Zangen A. Three-dimensional distribution of the electric field induced in the brain by transcranial magnetic stimulation using figure-8 and deep H-coils. J Clin Neurophysiol. 2007;24(1):31–8. doi: 10.1097/WNP.0b013e31802fa393 17277575 10.1097/WNP.0b013e31802fa393

[19] Tang A, Thickbroom G, Rodger J. Repetitive Transcranial Magnetic Stimulation of the Brain: Mechanisms from Animal and Experimental Models. Neuroscientist. 2015 doi: 10.1177/1073858415618897 . 26643579 10.1177/1073858415618897

[20] Dorward NL, Paleologos TS, Alberti O, Thomas DGT. The advantages of frameless stereotactic biopsy over frame-based biopsy. Brit J Neurosurg. 2002;16(2):110–8. doi: 10.1080/02688690220131705 12046728 10.1080/02688690220131705

[21] Muller PA, Dhamne SC, Vahabzadeh-Hagh AM, Pascual-Leone A, Jensen FE, Rotenberg A. Suppression of Motor Cortical Excitability in Anesthetized Rats by Low Frequency Repetitive Transcranial Magnetic Stimulation. PloS one. 2014;9(3). ARTN e91065 doi: 10.1371/journal.pone.0091065 24646791 10.1371/journal.pone.0091065 PMC3960125

[22] Waelbers T, Peremans K, Vermeire S, Duchateau L, Audenaert K, Dobbeleir A, et al Medetomidine causes changes in regional cerebral blood flow measured with single photon emission computed tomography in dogs. European journal of nuclear medicine and molecular imaging. 2009;36:S469–S.

[23] Waelbers T, Polis I, Vermeire S, Dobbeleir A, Eersels J, De Spiegeleer B, et al Effect of ketamine on the regional cerebral blood flow and binding index of the 5-HT2A receptor radioligand I-123-R91150 in the canine brain. J Vet Behav. 2015;10(4):332–7. doi: 10.1016/j.jveb.2015.03.009

[24] Newberg LA, Milde JH, Michenfelder JD. The Cerebral Metabolic Effects of Isoflurane at and above Concentrations That Suppress Cortical Electrical-Activity. Anesthesiology. 1983;59(1):23–8. doi: 10.1097/00000542-198307000-00005 6859608 10.1097/00000542-198307000-00005

[25] Valero-Cabre A, Payne BR, Rushmore J, Lomber SG, Pascual-Leone A. Impact of repetitive transcranial magnetic stimulation of the parietal cortex on metabolic brain activity: a 14C-2DG tracing study in the cat. Experimental brain research. 2005;163(1):1–12. doi: 10.1007/s00221-004-2140-6 . 15688174 10.1007/s00221-004-2140-6

[26] Moliadze V, Zhao Y, Eysel U, Funke K. Effect of transcranial magnetic stimulation on single-unit activity in the cat primary visual cortex. J Physiol. 2003;553(Pt 2):665–79. doi: 10.1113/jphysiol.2003.050153 ; PubMed Central PMCID: PMCPMC2343567. 12963791 10.1113/jphysiol.2003.050153 PMC2343567

[27] Ohnishi T, Hayashi T, Okabe S, Nonaka I, Matsuda H, Iida H, et al Endogenous dopamine release induced by repetitive transcranial magnetic stimulation over the primary motor cortex: an [11C]raclopride positron emission tomography study in anesthetized macaque monkeys. Biological psychiatry. 2004;55(5):484–9. doi: 10.1016/j.biopsych.2003.09.016 . 15023576 10.1016/j.biopsych.2003.09.016

[28] Amaya F, Paulus W, Treue S, Liebetanz D. Transcranial magnetic stimulation and PAS-induced cortical neuroplasticity in the awake rhesus monkey. Clinical Neurophysiology. 2010;121(12):2143–51. doi: 10.1016/j.clinph.2010.03.058 20570557 10.1016/j.clinph.2010.03.058

[29] Valero-Cabre A, Payne BR, Pascual-Leone A. Opposite impact on 14C-2-deoxyglucose brain metabolism following patterns of high and low frequency repetitive transcranial magnetic stimulation in the posterior parietal cortex. Experimental brain research. 2007;176(4):603–15. doi: 10.1007/s00221-006-0639-8 . 16972076 10.1007/s00221-006-0639-8

[30] De Decker S, Van Soens I, Duchateau L, Gielen IM, van Bree HJ, Binst DH, et al Transcranial magnetic stimulation in Doberman Pinschers with clinically relevant and clinically irrelevant spinal cord compression on magnetic resonance imaging. Journal of the American Veterinary Medical Association. 2011;238(1):81–8. doi: 10.2460/javma.238.1.81 . 21194326 10.2460/javma.238.1.81

[31] Amendt HL, Siedenburg JS, Steffensen N, Sobbeler FJ, Schutter A, Tunsmeyer J, et al Transcranial magnetic stimulation with acepromazine or dexmedetomidine in combination with levomethadone/fenpipramide in healthy Beagle dogs. Vet J. 2016;217:40–2. doi: 10.1016/j.tvjl.2016.06.006 27810209 10.1016/j.tvjl.2016.06.006

[32] Salinas FS, Szabo CA, Zhang W, Jones L, Leland MM, Wey HY, et al Functional neuroimaging of the baboon during concurrent image-guided transcranial magnetic stimulation. Neuroimage. 2011;57(4):1393–401. doi: 10.1016/j.neuroimage.2011.05.065 ; PubMed Central PMCID: PMCPMC3139451. 21664276 10.1016/j.neuroimage.2011.05.065 PMC3139451

[33] Shively CA, Clarkson TB. The Unique Value of Primate Models in Translational Research. Am J Primatol. 2009;71(9):715–21. doi: 10.1002/ajp.20720 19507247 10.1002/ajp.20720

[34] Eren I, Tukel R, Polat A, Karaman R, Unal S. Evaluation of regional cerebral blood flow changes in panic disorder with Tc99m-HMPAO SPECT. Psychiatry research. 2003;123(2):135–43. . 12850252 10.1016/s0925-4927(03)00062-3

[35] Vermeire S, Audenaert K, Dobbeleir A, De Meester R, Vandermeulen E, Waelbers T, et al Regional Cerebral Blood Flow Changes in Dogs with Anxiety Disorders, Measured with SPECT. Brain Imaging Behav. 2009;3(4):342–9. doi: 10.1007/s11682-009-9076-1

[36] Vermeire ST, Audenaert KR, Dobbeleir AA, De Meester RH, De Vos FJ, Peremans KY. Evaluation of the Brain 5-HT2A Receptor Binding Index in Dogs with Anxiety Disorders, Measured with (123)I-5I-R91150 and SPECT. J Nucl Med. 2009;50(2):284–9. doi: 10.2967/jnumed.108.055731 19164223 10.2967/jnumed.108.055731

[37] Irimajiri M, Miller MA, Green MA, Jaeger CB, Luescher AU, Hutchins GD. Cerebral metabolism in dogs assessed by (18)F-FDG PET: a pilot study to understand physiological changes in behavioral disorders in dogs. The Journal of veterinary medical science / the Japanese Society of Veterinary Science. 2010;72(1):1–6. . 19861888 10.1292/jvms.09-0048

[38] Baeken C, Marinazzo D, Wu GR, Van Schuerbeek P, De Mey J, Marchetti I, et al Accelerated HF-rTMS in treatment-resistant unipolar depression: Insights from subgenual anterior cingulate functional connectivity. World J Biol Psychia. 2014;15(4):286–97. doi: 10.3109/15622975.2013.872295 24447053 10.3109/15622975.2013.872295

[39] Adriaens A, Polis I, Waelbers T, Vandermeulen E, Dobbeleir A, De Spiegeleer B, et al NORMAL REGIONAL DISTRIBUTION OF CEREBRAL BLOOD FLOW IN DOGS: COMPARISON BETWEEN Tc-99m-ETHYLCYSTEINATE DIMER AND Tc-99m- HEXAMETHYLPROPYLENE AMINE OXIME SINGLE PHOTON EMISSION COMPUTED TOMOGRAPHY. Veterinary Radiology & Ultrasound. 2013;54(4):403–7. doi: 10.1111/vru.12028 23496105 10.1111/vru.12028

[40] LeBlanc AK, Peremans K. PET and SPECT Imaging in Veterinary Medicine. Semin Nucl Med. 2014;44(1):47–56. doi: 10.1053/j.semnuclmed.2013.08.004 24314045 10.1053/j.semnuclmed.2013.08.004

[41] Dockx R, Baeken C, Duprat R, De Vos F, De Spiegeleer B, Dobbeleir A, et al Regional cerebral blood flow changes after accelerated repetitive transcranial magnetic stimulation of the canine frontal cortex. European journal of nuclear medicine and molecular imaging. 2015;42:S290–S.

[42] Dockx R, Baeken C, Duprat R, De Vos F, De Spiegeleer B, Dobbeleir A, et al Proof of concept study on the increased perfusion at stimulation site after Accelerated HF-rTMS over the canine's left frontal cortex. in submission.

[43] Dockx R, Peremans K, Duprat R, Vlerick L, Van Laeken N, Saunders JH, et al Accurate external localization of the left frontal cortex in dogs by using pointer based frameless neuronavigation. PeerJ. 2017;Accepted for publication 10.7717/peerj.3425 PMC5507169 28713649

[44] Baeken C, Vanderhasselt MA, Remue J, Herremans S, Vanderbruggen N, Zeeuws D, et al Intensive HF-rTMS treatment in refractory medication-resistant unipolar depressed patients. Journal of affective disorders. 2013;151(2):625–31. doi: 10.1016/j.jad.2013.07.008 23896317 10.1016/j.jad.2013.07.008

[45] Baeken C, Marinazzo D, Everaert H, Wu GR, Van Hove C, Audenaert K, et al The Impact of Accelerated HF-rTMS on the Subgenual Anterior Cingulate Cortex in Refractory Unipolar Major Depression: Insights From (18)FDG PET Brain Imaging. Brain stimulation. 2015;8(4):808–15. doi: 10.1016/j.brs.2015.01.415 . 25744500 10.1016/j.brs.2015.01.415

[46] Teneback CC, Nahas Z, Speer AM, Molloy M, Stallings LE, Spicer KM, et al Changes in prefrontal cortex and paralimbic activity in depression following two weeks of daily left prefrontal TMS. J Neuropsych Clin N. 1999;11(4):426–35. 10.1176/jnp.11.4.426 10570754

[47] Catafau AM, Perez V, Gironell A, Martin JC, Kulisevsky J, Estorch M, et al SPECT mapping of cerebral activity changes induced by repetitive transcranial magnetic stimulation in depressed patients. A pilot study. Psychiat Res-Neuroim. 2001;106(3):151–60. doi: 10.1016/S0925-4927(01)00079-8 10.1016/s0925-4927(01)00079-8 11382537

[48] O'Reardon JP, Solvason HB, Janicak PG, Sampson S, Isenberg KE, Nahas Z, et al Efficacy and safety of transcranial magnetic stimulation in the acute treatment of major depression: a multisite randomized controlled trial. Biological psychiatry. 2007;62(11):1208–16. doi: 10.1016/j.biopsych.2007.01.018 . 17573044 10.1016/j.biopsych.2007.01.018

[49] Gersner R, Kravetz E, Feil J, Pell G, Zangen A. Long-Term Effects of Repetitive Transcranial Magnetic Stimulation on Markers for Neuroplasticity: Differential Outcomes in Anesthetized and Awake Animals. Journal of Neuroscience. 2011;31(20):7521–6. doi: 10.1523/JNEUROSCI.6751-10.2011 21593336 10.1523/JNEUROSCI.6751-10.2011 PMC6622610

[50] Knoch D, Treyer V, Regard M, Müri RM, Buck A, Weber B. Lateralized and frequency-dependent effects of prefrontal rTMS on regional cerebral blood flow. NeuroImage. 2006;31(2):641–8. doi: http://dx.doi.org/10.1016/j.neuroimage.2005.12.025 16497518 10.1016/j.neuroimage.2005.12.025

[51] Richieri R, Boyer L, Farisse J, Colavolpe C, Mundler O, Lancon C, et al Predictive value of brain perfusion SPECT for rTMS response in pharmacoresistant depression. European journal of nuclear medicine and molecular imaging. 2011;38(9):1715–22. doi: 10.1007/s00259-011-1850-9 21647787 10.1007/s00259-011-1850-9

[52] Massimini M, Ferrarelli F, Huber R, Esser SK, Singh H, Tononi G. Breakdown of cortical effective connectivity during sleep. Science. 2005;309(5744):2228–32. doi: 10.1126/science.1117256 16195466 10.1126/science.1117256

[53] Ferrarelli F, Massimini M, Sarasso S, Casali A, Riedner BA, Angelini G, et al Breakdown in cortical effective connectivity during midazolam-induced loss of consciousness. Proceedings of the National Academy of Sciences of the United States of America. 2010;107(6):2681–6. doi: 10.1073/pnas.0913008107 20133802 10.1073/pnas.0913008107 PMC2823915

[54] Avery DH, Holtzheimer PE 3rd, Fawaz W, Russo J, Neumaier J, Dunner DL, et al A controlled study of repetitive transcranial magnetic stimulation in medication-resistant major depression. Biological psychiatry. 2006;59(2):187–94. doi: 10.1016/j.biopsych.2005.07.003 . 16139808 10.1016/j.biopsych.2005.07.003

[55] Rumi DO, Gattaz WF, Rigonatti SP, Rosa MA, Fregni F, Rosa MO, et al Transcranial magnetic stimulation accelerates the antidepressant effect of amitriptyline in severe depression: A double-blind placebo-controlled study. Biological psychiatry. 2005;57(2):162–6. doi: 10.1016/j.biopsych.2004.10.029 15652875 10.1016/j.biopsych.2004.10.029

[56] George MS, Stallings LE, Speer AM, Nahas Z, Spicer KM, Vincent DJ, et al Prefrontal repetitive transcranial magnetic stimulation (rTMS) changes relative perfusion locally and remotely. Hum Psychopharm Clin. 1999;14(3):161–+. doi: 10.1002/(Sici)1099-1077(199904)14:3<161::Aid-Hup73>3.0.Co;2–2

[57] Brakemeier EL, Wilbertz G, Rodax S, Danker-Hopfe H, Zinka B, Zwanzger P, et al Patterns of response to repetitive transcranial magnetic stimulation (rTMS) in major depression: Replication study in drug-free patients. Journal of affective disorders. 2008;108(1–2):59–70. doi: 10.1016/j.jad.2007.09.007 17963846 10.1016/j.jad.2007.09.007

[58] Brakemeier EL, Luborzewski A, Danker-Hopfe H, Kathmann N, Bajbouj M. Positive predictors for antidepressive response to prefrontal repetitive transcranial magnetic stimulation (rTMS). Journal of Psychiatric Research. 2007;41(5):395–403. doi: 10.1016/j.jpsychires.2006.01.013 16554071 10.1016/j.jpsychires.2006.01.013

[59] Conelea CA, Philip NS, Yip AG, Barnes JL, Niedzwiecki MJ, Greenberg BD, et al Transcranial magnetic stimulation for treatment-resistant depression: Naturalistic treatment outcomes for younger versus older patients. Journal of affective disorders. 2017;217:42–7. doi: 10.1016/j.jad.2017.03.063 . 28388464 10.1016/j.jad.2017.03.063 PMC5460629

[60] Sabesan P, Lankappa S, Khalifa N, Krishnan V, Gandhi R, Palaniyappan L. Transcranial magnetic stimulation for geriatric depression: Promises and pitfalls. World J Psychiatry. 2015;5(2):170–81. doi: 10.5498/wjp.v5.i2.170 ; PubMed Central PMCID: PMCPMC4473489. 26110119 10.5498/wjp.v5.i2.170 PMC4473489

[61] Fregni F, Marcolin MA, Myczkowski M, Amiaz R, Hasey G, Rumi DO, et al Predictors of antidepressant response in clinical trials of transcranial magnetic stimulation. Int J Neuropsychoph. 2006;9(6):641–54. doi: 10.1017/S1461145705006280 16939662

[62] Levkovitz Y, Segal M. Aging affects transcranial magnetic modulation of hippocampal evoked potentials. Neurobiol Aging. 2001;22(2):255–63. doi: 10.1016/S0197-4580(00)00195-0 11182475 10.1016/s0197-4580(00)00195-0

[63] Su MY, Head E, Brooks WM, Wang Z, Muggenburg BA, Adam GE, et al Magnetic resonance imaging of anatomic and vascular characteristics in a canine model of human aging. Neurobiol Aging. 1998;19(5):479–85. . 9880050 10.1016/s0197-4580(98)00081-5

[64] Huang CC, Wei IH, Chou YH, Su TP. Effect of age, gender, menopausal status, and ovarian hormonal level on rTMS in treatment-resistant depression. Psychoneuroendocrino. 2008;33(6):821–31. doi: 10.1016/j.psyneuen.2008.03.006 18468810 10.1016/j.psyneuen.2008.03.006

[65] Lisanby SH, Gutman D, Luber B, Schroeder C, Sackeim HA. Sham TMS: Intracerebral measurement of the induced electrical field and the induction of motor-evoked potentials. Biological psychiatry. 2001;49(5):460–3. doi: 10.1016/S0006-3223(00)01110-0 11274658 10.1016/s0006-3223(00)01110-0

