# Supplementary figures and images for: Anaesthesia, not number of sessions, influences the magnitude and duration of an aHF-rTMS in dogs
Source: PLoS One. 2017 Sep 22;12(9):e0185362. doi: 10.1371/journal.pone.0185362 (PMC5609759; doi:10.1371/journal.pone.0185362)

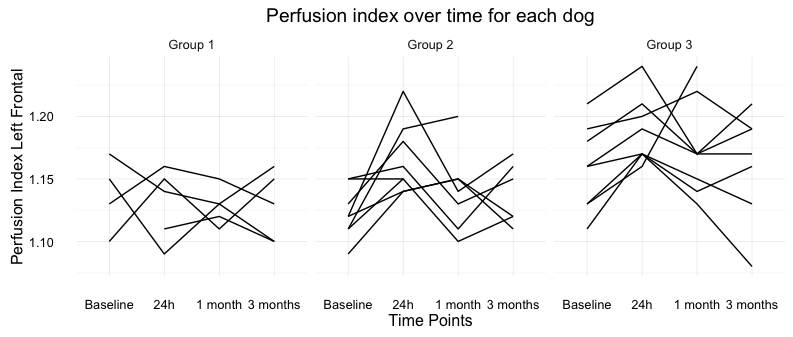

Supplement: S1 Fig — (TIF) [file pone.0185362.s001.tif]

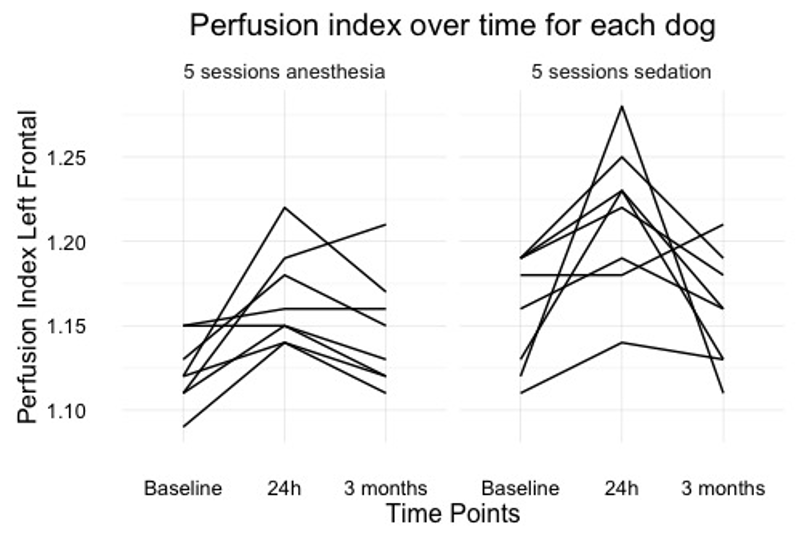

Supplement: S2 Fig — (TIF) [file pone.0185362.s002.tif]

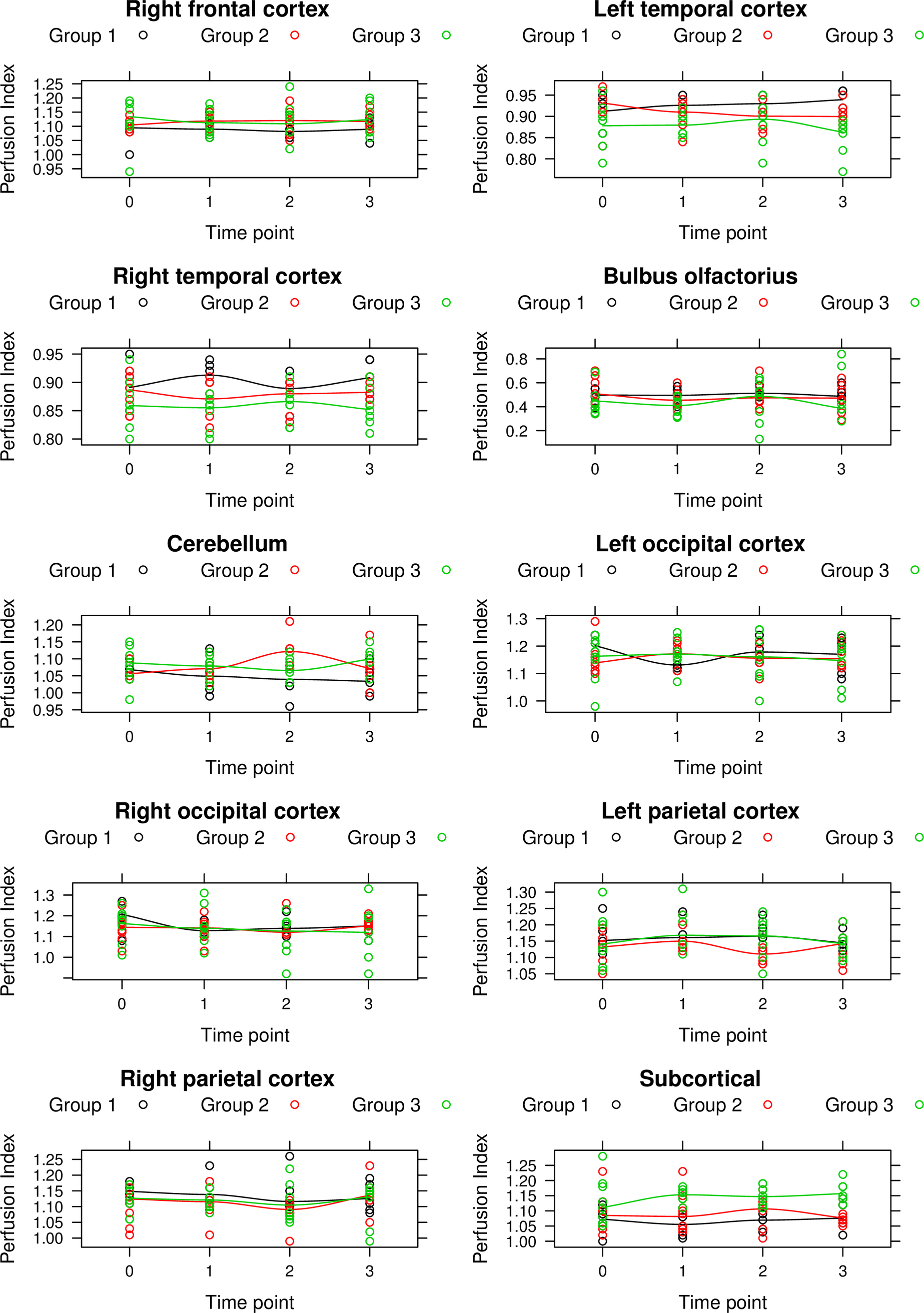

Supplement: S3 Fig — (TIF) [file pone.0185362.s003.tif]

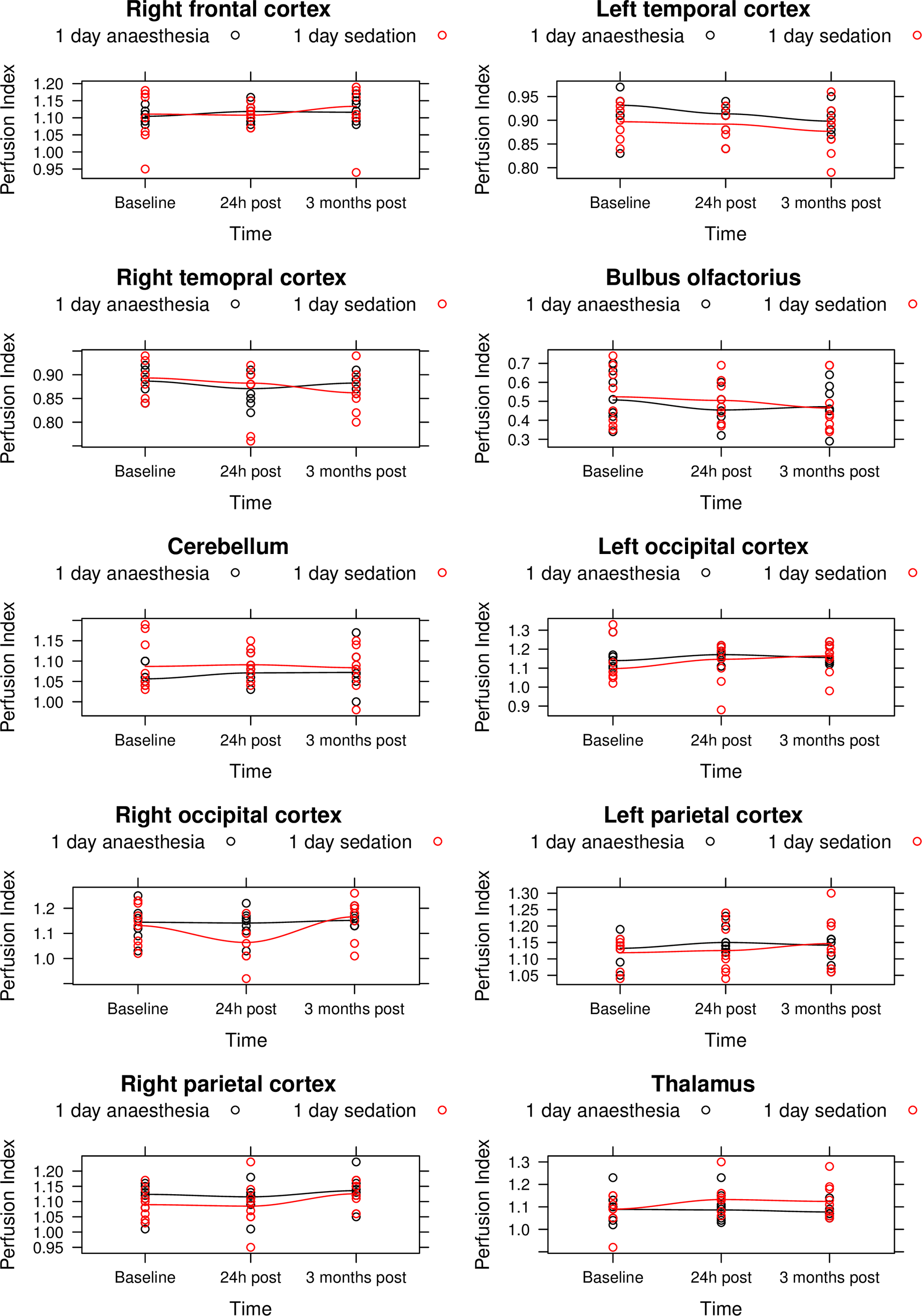

Supplement: S4 Fig — (TIF) [file pone.0185362.s004.tif]

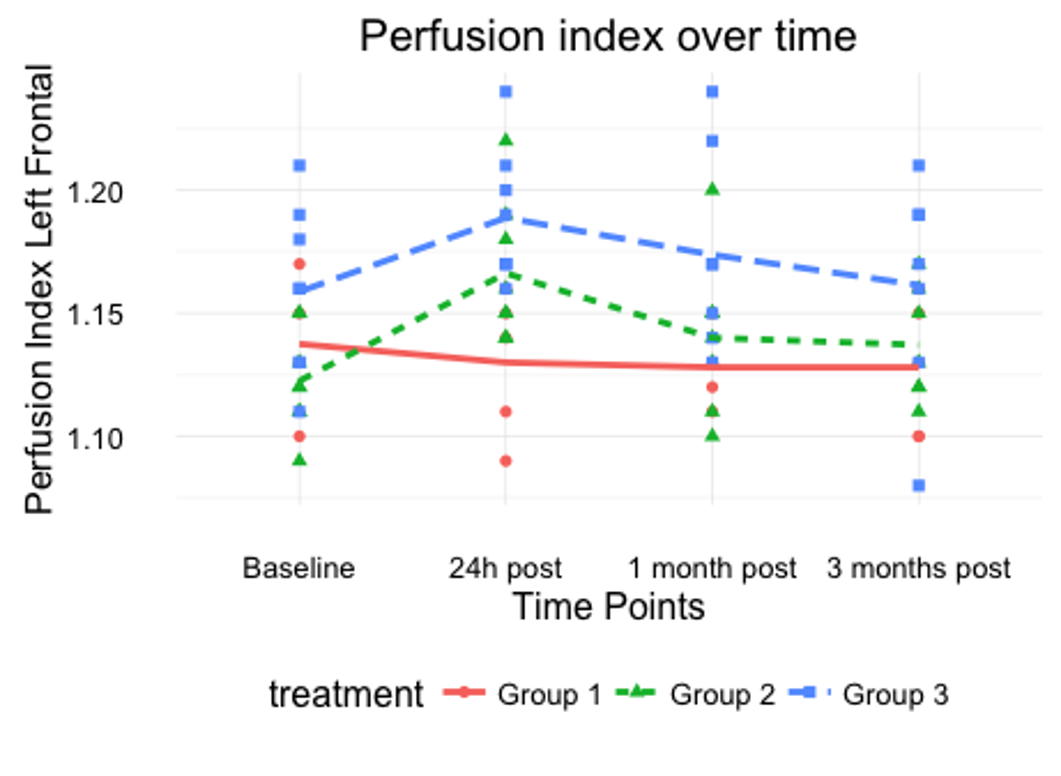

Supplement: S5 Fig — (TIF) [file pone.0185362.s005.tif]

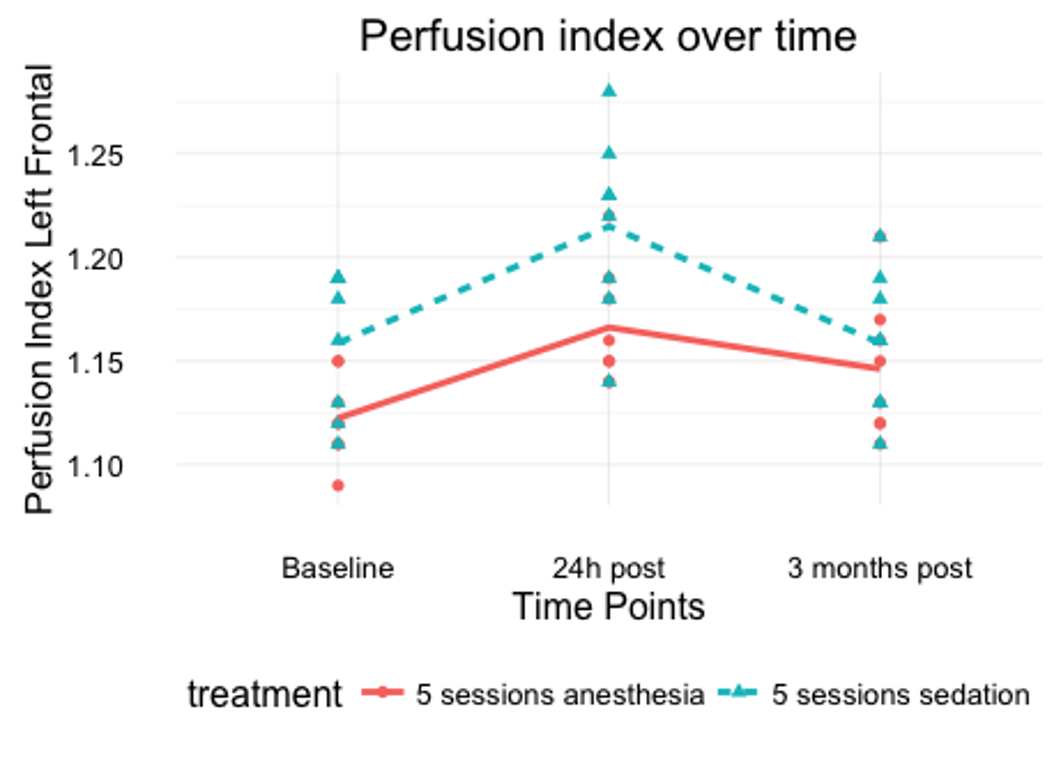

Supplement: S6 Fig — (TIF) [file pone.0185362.s006.tif]
