# Supplementary material for: Anaesthesia, not number of sessions, influences the magnitude and duration of an aHF-rTMS in dogs
Source: PLoS One. 2017 Sep 22;12(9):e0185362. doi: 10.1371/journal.pone.0185362 (PMC5609759; doi:10.1371/journal.pone.0185362)
Supplement: S2 Table — (DOCX) [file pone.0185362.s008.docx]

|  |  | **Baseline** | | | |  | | **24 hours post** | | | |  | | **3 months post** | | | |  |
| --- | --- | --- | --- | --- | --- | --- | --- | --- | --- | --- | --- | --- | --- | --- | --- | --- | --- | --- |
|  | | *Mean* | *SD* | *Min* | *Max* | | *Mean* | | *SD* | *Min* | *Max* | | *Mean* | | *SD* | *Min* | *Max* | |
| **Group 2** (n=8) | | 1.12 | 0.02 | 1.09 | 1.15 | | 1.17 | | 0.03 | 1.14 | 1.22 | | 1.15 | | 0.03 | 1.11 | 1.21 | |
| Males | | 1.12 | 0.02 | 1.09 | 1.15 | | 1.17 | | 0.03 | 1.14 | 1.22 | | 1.16 | | 0.03 | 1.11 | 1.21 | |
| Females | | 1.14 | 0.01 | 1.12 | 1.15 | | 1.15 | | 0.01 | 1.14 | 1.15 | | 1.12 | | 0.00 | 1.12 | 1.12 | |
| ]2y-4y] | | 1.15 | 0.03 | 1.13 | 1.18 | | 1.18 | | *NA* | 1.18 | 1.18 | | 1.15 | | *NA* | 1.15 | 1.15 | |
| ]4y-6y] | | *NA* | *NA* | *NA* | *NA* | | *NA* | | *NA* | *NA* | *NA* | | *NA* | | *NA* | *NA* | *NA* | |
| ]6y-8y] | | 1.14 | 0.03 | 1.11 | 1.19 | | 1.17 | | 0.02 | 1.15 | 1.19 | | 1.17 | | 0.04 | 1.13 | 1.21 | |
| **Group 4** (n=8) | | 1.16 | 0.03 | 1.11 | 1.19 | | 1.21 | | 0.04 | 1.14 | 1.28 | | 1.16 | | 0.03 | 1.11 | 1.21 | |
| Males | | 1.15 | 0.03 | 1.11 | 1.18 | | 1.19 | | 0.04 | 1.14 | 1.23 | | 1.16 | | 0.04 | 1.13 | 1.21 | |
| Females | | 1.20 | 0.05 | 1.12 | 1.28 | | 1.25 | | 0.03 | 1.22 | 1.28 | | 1.16 | | 0.04 | 1.11 | 1.19 | |
| ]2y-4y] | | *NA* | *NA* | *NA* | *NA* | | *NA* | | *NA* | *NA* | *NA* | | *NA* | | *NA* | *NA* | *NA* | |
| ]4y-6y] | | 1.2 | 0.03 | 1.16 | 1.25 | | 1.19 | | 0.03 | 1.16 | 1.21 | | 1.19 | | 0.03 | 1.16 | 1.21 | |
| ]6y-8y] | | 1.14 | 0.03 | 1.11 | 1.19 | | 1.21 | | 0.05 | 1.14 | 1.28 | | 1.14 | | 0.03 | 1.11 | 1.18 | |
